# Optical sectioning methods in three-dimensional bioimaging

**DOI:** 10.1038/s41377-024-01677-x

**Published:** 2025-01-01

**Authors:** Jing Zhang, Wei Qiao, Rui Jin, Hongjin Li, Hui Gong, Shih-Chi Chen, Qingming Luo, Jing Yuan

**Affiliations:** 1 Britton Chance Center for Biomedical Photonics, Wuhan National Laboratory for Optoelectronics, Huazhong University of Science and Technology, Wuhan, China; 2 MoE Key Laboratory for Biomedical Photonics, Innovation Institute, Huazhong University of Science and Technology, Wuhan, China; 3 Hong Kong Center for Cerebro-Cardiovascular Health Engineering, N.T, Hong Kong, China; 4 Department of Biomedical Engineering, City University of Hong Kong, Hong Kong, China; 5 HUST-Suzhou Institute for Brainsmatics, Suzhou, China; 6 Department of Mechanical and Automation Engineering, The Chinese University of Hong Kong, Shatin, N.T., Hong Kong, China; 7 School of Biomedical Engineering, Hainan University, Haikou, China

**Keywords:** Biophotonics, Microscopy

## Abstract

In recent advancements in life sciences, optical microscopy has played a crucial role in acquiring high-quality three-dimensional structural and functional information. However, the quality of 3D images is often compromised due to the intense scattering effect in biological tissues, compounded by several issues such as limited spatiotemporal resolution, low signal-to-noise ratio, inadequate depth of penetration, and high phototoxicity. Although various optical sectioning techniques have been developed to address these challenges, each method adheres to distinct imaging principles for specific applications. As a result, the effective selection of suitable optical sectioning techniques across diverse imaging scenarios has become crucial yet challenging. This paper comprehensively overviews existing optical sectioning techniques and selection guidance under different imaging scenarios. Specifically, we categorize the microscope design based on the spatial relationship between the illumination and detection axis, i.e., on-axis and off-axis. This classification provides a unique perspective to compare the implementation and performances of various optical sectioning approaches. Lastly, we integrate selected optical sectioning methods on a custom-built off-axis imaging system and present a unique perspective for the future development of optical sectioning techniques.

## Introduction

Over the years, the understanding of biology has deepened from simple cells to complex organisms thanks to the advancement in optical microscopy. It benefits from the enhanced capability to acquire high-quality three-dimensional (3D) structural and functional information at a sub-micron level resolution. However, substantial out-of-focus fluorescent backgrounds often compromise the image quality. Various methods for optical sectioning have been explored and developed to mitigate the impact from the background while preserving in-focus details and the sample’s integrity to address this issue.

While the most common obstacle in optical imaging from the intense out-of-focus fluorescent background has been gradually resolved over the years, the biological complexity presents different challenges. Specifically, the imaging area ranges from cellular scale to organ- and tissue-scale, while determining the delicate structures of sub-micrometers and the functional units of millimeters is required. Furthermore, the high dynamic range of labeled signals poses unique challenges. Additionally, there is a need to address the depth of penetration of illumination for scattering specimens such as the whole mammalian brain. Although numerous optical sectioning techniques have been developed to meet different needs, a well-rounded selection guidance for these techniques is still essential to meet the specific demands under various imaging scenarios, or the lack of which can cause suboptimal performance and higher time costs when transitioning to a different imaging scenario without adopting a compatible imaging method.

Confocal microscopy, the most commonly used optical sectioning method in imaging fixed cells, is deemed the norm in biological imaging^1^. However, the high phototoxicity and insufficient penetration depth prevent it from applications in living imaging. As biologists explore functionality beyond structural features, the penetration depth of the illumination beam also needs to go deeper into the target area. While nonlinear microscopy offers superior penetration depth due to multi-photon effects^2^, confocal and nonlinear microscopy suffer from low temporal resolution, hindering the extension of exploratory scope beyond the cellular level from including more complex biological tissues. Hence, alternative methods such as spinning disk and line confocal microscopy (LC) have been developed^3,4^. Nevertheless, these methods increase the imaging speed through parallel scanning at the expense of reduced optical sectioning strength, which results in lower image quality.

The introduction of structured illumination microscopy (SIM), capable of achieving a balance of optical sectioning strength and imaging speed, has enabled the imaging of complete tissue structures, thus revolutionizing wide-field optical sectioning^5^. However, repetitive captures in SIM aggravate phototoxicity and prevent the camera from reaching its maximum throughput. In recent decades, light sheet microscopy has been developed for its capability to perform selective illumination that enables optical sectioning in a single snapshot^6^. With low phototoxicity and high imaging rate, light sheet microscopy has found valuable applications in more biological domains, such as embryonic development.

Despite the continuous progress in optical sectioning techniques, imaging thick tissues and large samples remains challenging. The image quality is often less than ideal due to diminished optical sectioning strength caused by factors such as scattering. Single-scan optical sectioning microscopy, e.g., line-illumination modulation microscopy (LiMo)^7^, has recently been introduced. These methods attain an ideal balance between the speed and throughput via line scanning imaging methods and preserve superior optical sectioning strength than the original data via post-processing reconstruction algorithms, which allow for acceptable imaging at the organ level.

With recent developments in optical sectioning methods, a comprehensive review and selection guidance to identify the optimal method under different application scenarios becomes imperative. This paper aims to study and assess different optical sectioning methods based on their respective principles. We first categorize existing optical sectioning methods into coaxial (i.e., on-axis) and off-axis imaging based on the spatial relationship between the illumination and detection axes. In each category, we review the recent development of various optical sectioning techniques, comprehensively compare their imaging performance, and summarize their respective advantages and potential application scenarios. Finally, we demonstrate various optical sectioning methods within the same system via off-axis imaging and offer insight into the future development of optical sectioning technology.

## Optical sectioning methods

Current optical imaging methods employ epi-illumination for its simplicity and ease of implementation. However, in most epi-illumination setups, the illumination and detection axes overlap, causing the detector to pick up all signals out of focus as background. Nevertheless, combined with various demodulation methods, optical sectioning can be achieved (e.g., confocal, two-photon, SIM microscopy). Another revolutionary strategy improves optical systems by separating illumination from detection axes, addressing epi-illumination drawbacks. The system setup naturally blocks out-of-focus (e.g., light sheet microscopy) signals or in-focus (e.g., LiMo microscopy) signals, depending on the alignment of illumination and detection axes.

**Fig. 1:**
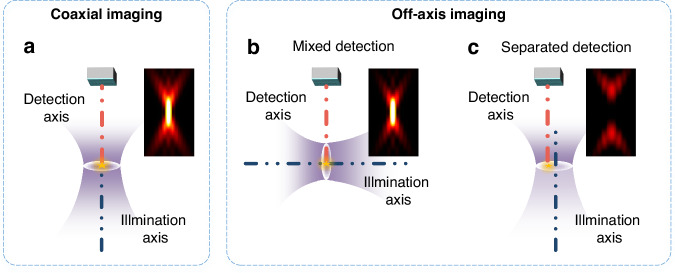
Schematic diagram of coaxial and off-axis imaging. **a** Schematic diagram of coaxial imaging. **b** Schematic diagram of mixed detection in off-axis imaging. **c** Schematic diagram of separated detection in off-axis imaging. Images beside each system diagram represent the point spread function along the xz direction

Therefore, based on the assumption that the sample is a single ideal point in the center of the field of view, we can divide these methods into coaxial and off-axis imaging according to the location between the illumination and detection axes. Coaxial imaging signifies that the illumination and detection axes coincide (Fig. 1a), which results in a high degree of fusion of the in-focus and out-of-focus signals. Off-axis imaging indicates that the illumination and the detection axes have a specific offset or angle. As shown in Fig. 1b and c, in-focus and out-of-focus information are better distinguished than coaxial imaging, achieving better optical sectioning strength. It is noted that SIM is considered off-axis imaging with phase shifts in a strict definition. However, in practical imaging, uniform bias surpasses sinusoidal modulation due to background or blurring. Thus, raw images of SIM exhibit wide-field characteristics, classifying SIM as coaxial imaging.

To analyze quantitatively, we assess the ability of optical sectioning by its axial response to the thin fluorescent sheet. For example, a faster decay in the intensity along the defocusing direction indicates a more substantial background suppression capability. The system’s optical transfer function (OTF) can be obtained by the Fourier transform of the point spread function (PSF). Therefore, the optical sectioning strength can be expressed as the full width at half maximum (FWHM) in Eq. (1)^8^.

1 $$I(u)=F[({S}_{eff}\times {D}_{eff})\otimes 1]$$

where *u*, *I*, *S*_*eff*_, and *D*_*eff*_ are the axial optical coordinate, detected image, illumination PSF, and detection PSF, respectively. For conventional wide-field microscopy, the illumination is uniform, and the detection is not constrained, which means the imaging characteristics are determined by the PSF of the objective lens, *h*_*eff*._ As expressed in Eq. (1), the defocusing component does not decay with the degree of defocusing, indicating a lack of optical sectioning capability in wide-field microscopy.

**Fig. 2:**
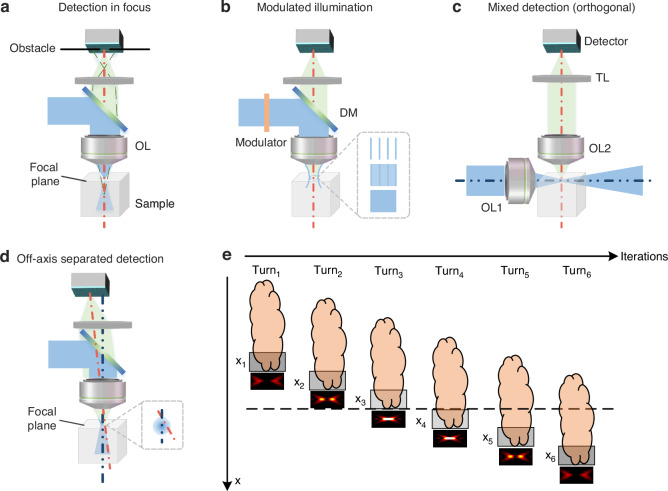
Schematic diagram of optical sectioning methods. **a** Focal plane conjugation in coaxial imaging. **b** Modulated illumination in coaxial imaging. **c** Mixed detection in off-axis imaging. **d** Separated detection in off-axis imaging. **e** Multiple scanning of separated detection

**Fig. 3:**
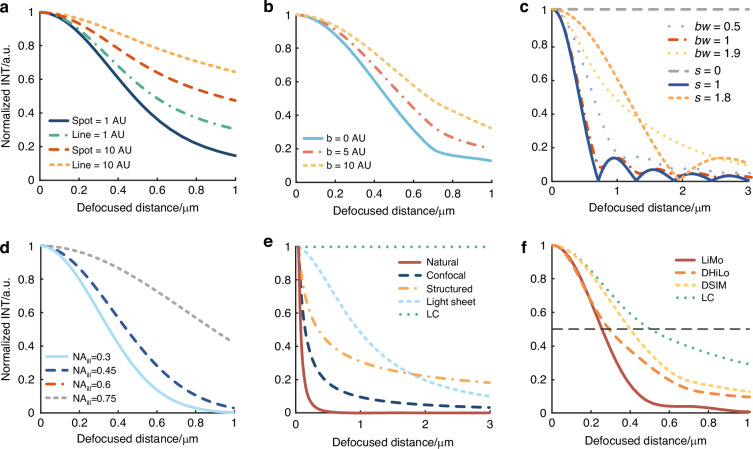
Optical sectioning strength curves for various sectioning methods. **a** Optical sectioning strength curves for focal plane conjugation. **b** Optical sectioning strength curves for intra-focal excitation. **c** Optical sectioning strength curves for modulated illumination, where *s*_*max*_ is set to 2, and ‘*bw*’ represents the filter bandwidth. **d** Optical sectioning strength curves for mixed detection. **e** Background suppressions in thick samples for various sectioning methods. **f** Optical sectioning strength for various reconstruction methods in separated detection

For coaxial imaging and off-axis imaging optical sectioning methods, diverse constraints have to be added to the basic model to predict the sectioning strength. Further details of these methods are provided in the following sections. Figures 2 and 3 show their schematic diagrams and optical sectioning strength curves.

### Coaxial imaging

Focal plane conjugation, intra-focal excitation, and modulated illumination are three primary optical sectioning methods in a coaxial imaging system. By changing the illumination or detection PSFs in the model, the defocused component’s intensity can be reduced efficiently, allowing for extracting focal plane information from the highly mixed in-focus defocus information.

#### Focal plane conjugation

For focal plane conjugation detection, the emission is excited by a spot or line-shaped illumination and then blocked by a small hole or slit of limited size in front of the detector (Fig. 2a). When defocused, the detected signal is rapidly divergent. Thus, the small hole or slit in front of the detector can effectively block the divergent signal to enhance the optical sectioning strength.

In the presence of a physical obstacle, the function of axial response to judge optical sectioning can be expressed as^9^

2 $${I}_{Confocal}(u)=\left\{\begin{array}{l}\displaystyle\int jinc(bs){T}^{2}(s,u)jinc(\beta s)sds\,{\rm{Pinhole}}\\ \displaystyle\int \sin c(b{s}_{x}/\pi ){T}^{2}({s}_{x},0,u)\sin c(\beta {s}_{x}/\pi )d{s}_{x}\,{\rm{Slit}}\end{array}\right.$$

where *b* represents the radius of the illumination spot, *β* represents the radius of the pinhole or slit at the detection end, *s* is the coordinate in the frequency domain, and *u* represents optical defocus distance, which is related to the defocus distance, the wavelength of illumination and detection, and the numerical aperture (NA) of the objective. *T* is the OTF of the system, i.e., the Fourier transform of the PSF heff, which also represents the system’s carrying capacity for different frequencies.

Due to physical constraints, *I(u)* is no longer a constant; thus, the system has optical sectioning capability^10^. Based on the size and shape of the obstacles, the curves indicating the decay of signal strength with defocusing distance can be plotted (Fig. 3a). The holes exhibit better optical sectioning strength than slits, and the smaller the size, the greater the optical sectioning strength. We described the two types of confocal microscopy systems, i.e., point confocal and line confocal, as “confocal” and “LC”, respectively.

#### Intra-focal excitation

In the case of intra-focal excitation methods, the illumination is confined to a short focal distance, preventing the excitation of signals beyond this range and extracting only the in-focus signals. Due to the illumination light concentrated exclusively within the focal plane, there is insufficient energy for nonlinear effects to excite out-of-focus signals. The optical system is the same as Fig. 2a, except for the absence of obstacles in front of the detector and different excitation sources.

The imaging process involving nonlinear effects is equivalent to a limited spot at the illumination and an ideal pinhole at the detection to simulate intra-focal excitation. We can express the optical sectioning capability as follows^11^:

3 $${I}_{Two-photon}(u)=\int jinc(2bs){T}^{2}(s,u/2)sds$$

where *b* represents the radius of the illumination spot. We can observe that the intensity response decreases with defocusing, which indicates the system’s optical sectioning capability. In multi-photon microscopy, adding a pinhole at the detection end can further enhance the optical sectioning capability. The optical sectioning strength increases with decreasing pinhole sizes (Fig. 3b).

#### Modulated illumination

Employing non-uniform illumination patterns on a two-dimensional (2D) plane is the key to the modulated illumination-based optical sectioning method. The high-frequency components in non-uniform illumination rapidly degrade into uniform illumination as defocusing occurs (Fig. 2b), which provides a basis for extracting in-focus signals after demodulating.

In the physical model, only high-frequency components in illumination serve as effective illumination, while the demodulation algorithm eliminates uniform illumination. The decay of modulation frequency with defocusing determines the optical sectioning strength. Therefore, after demodulation, the extracted information is limited to the depth affected by the modulated illumination. We obtain Eq. (4) to assess the optical sectioning strength in both structured and hybrid illuminations^12,13^.

4 $${I}_{Modulated}(u)=\sqrt{{\int }_{s\,\min }^{s\,\max }{T}^{2}(s,u)T(s,0)ds}$$

where in structured illumination, *s*_*max*_ = *s*_*min*_ = *s*_*0*_; in hybrid illumination, *s*_*max*_ and *s*_*min*_ depend on the extra filter in the demodulation algorithms. We define the filter’s bandwidth as the difference between *s*_*max*_ and *s*_*min*_.

Specifically, the intensity response variation with defocusing under different modulation frequencies in structured illumination and different filters in hybrid illumination is plotted (Fig. 3c), where *s*_*max*_ is set at 2 and ‘*bw*’ represents bandwidth. The optimal sectioning strengths of hybrid and structured illumination match half the cutoff frequency of conventional structured illumination. Moreover, hybrid illumination offers a tunable sectioning strength by adjusting the filter bandwidth.

The three methods mentioned above achieve optical sectioning by applying constraints at the illumination and detection ends. These methods can effectively distinguish in-focus and out-of-focus signals, which would be highly mixed in coaxial imaging.

### Off-axis imaging

In off-axis imaging systems, mixed and separated detection are the two main methods to distinguish in-focus and out-of-focus information directly. Unlike coaxial imaging methods (e.g., confocal microscopy), off-axis methods achieve strong optical sectioning in thick tissues through the non-coincidence of illumination and detection of optical axes. In coaxial imaging systems, the high overlap of in-focus and out-of-focus information hinders the extraction of signals in focus, resulting in poor optical sectioning performance in thick samples. On the other hand, off-axis imaging intrinsically suppresses the background and performs multiple mixing ratio detections for in-focus and out-of-focus information. Specifically, the significant change in in-focus information with an off-axis distance can realize various mixing ratios. In contrast, according to the system PSF, the out-of-focus information remains constant. Therefore, we can extract signals in focus, even in thick tissues with a high background. It should be noted that light sheet microscopy, despite its off-axis attributes, is constrained by the capability to capture only one single mixing ratio, thereby limiting its performance in thick samples, where the presence of scattering reduces optical sectioning strength. In theory, implementing optical sectioning methods in off-axis imaging systems can further improve imaging results.

#### Mixed detection

The off-axis mixed-detection method primarily relies on the perpendicular alignment of the illumination and detection axes, which constrains the illumination to a relatively small in-focus range in the detection interval (Fig. 2c). As opposed to the coaxial detection method, in the mixed detection system setup, a substantial portion of out-of-focus information remains unexcited. In contrast, the in-focus excitation is achieved directly to provide easy access to optical sectioning. However, due to the influence of tissue scattering, the effective excitation depth usually exceeds the ideal depth, causing a considerable amount of out-of-focus information in the mixed detection.

By the physical model, the illumination in mixed-detection merely excites a small depth covering the Rayleigh distance, determined by the NA of the objective lens used for illumination. A higher NA leads to a narrower illuminated range but a smaller field of view. Then, in the field of view, the expression of optical sectioning strength can be expressed in Eq. (5).

5 $${I}_{Lightsheet}(u)=\exp (-{u}^{2}/2{c}^{2})T({s}_{x},\,{s}_{y},\,u)\delta ({s}_{x},\,{s}_{y})$$

where *c* represents the radius of the illumination beam.

Specifically, as the illumination NA changes, the curve depicting the intensity response variation with defocus distance can be plotted in Fig. 3d. A higher NA of the illumination objective results in a thinner light sheet and stronger sectioning.

#### Separated detection

Separated detection uses multiple detectors positioned at different off-axis distances or angles. With the diverse degree of off-axis detection, the overall PSF is split into various distinct sub-PSFs. Once the defocusing occurs, the natural modulation based on the illumination PSF rapidly attenuates into uniform illumination, which allows for the extraction of in-focus signals.

Since high-frequency modulation in illumination is the critical factor for off-axis separated detection, the illumination spot must be focused at least in one dimension. In the focusing dimension, the illumination intensity is characterized by a Gaussian distribution. When the detector remains stationary with the sample, the shifting of the illumination spot allows a specific pixel in the sample to be illuminated by different parts of the 3D-varied illumination distribution caused by the natural illumination PSF. Therefore, separated detection enables the generation of distinctive illumination intensity modulations at various off-axis distances.

Due to the relative nature of illumination and detection, it is also possible to maintain the position of the illumination spot unchanged while altering the position of the detector. When both the sample and the detector move simultaneously and maintain a conjugate relationship, different angles between the illumination axis and the detection axis come into effect (Fig. 2d). As described previously, when the detector remains fixed at an off-axis position, the samples move across the entire imaging area. By adjusting the off-axis position and conducting multiple scans, we can acquire distinctively modulated signals, which enables the integration, encoding, and recording of spatial information from both in-focus and out-of-focus planes (Fig. 2e). In summary, in comparison with coaxial imaging that encounters axial information overlap, off-axis separated detection is more effective in extracting in-focus signals via different mixing ratios.

In the physical model, for the line-shaped illumination spot, the optical sectioning strength of the linear decoding reconstruction, LiMo, can be obtained using Eq. (6)^7^.

6 $${I}_{LiMo}(u)=\int \sin c(b{s}_{x}/\pi ){T}^{2}({s}_{x},0,u)\sin c(\beta {s}_{x}/\pi )[1-\,\cos (2\beta {s}_{x})]d{s}_{x}$$

where *b* represents the radius of the illumination spot, and *β* represents the radius of the slit.

To evaluate the background suppression via various methods in thick samples, responses from all defocused planes, e.g., from the defocus distance *u*_*1*_ to infinity, are integrated to estimate the influence of all deep-layer backgrounds deeper than *u*_*1*_. Then, the attenuation coefficients of defocused signals with defocus distance by different optical sectioning methods are obtained after simplifying and approximating the expression, as shown in Eq. (7)^14^.

7 $$\left\{\begin{array}{ll}{\displaystyle{\int} }_{{u}_{1}}^{\infty }{I}_{Natural}(u)du\propto {\displaystyle{\int} }_{{u}_{1}}^{\infty }{\displaystyle{\int} }_{0}^{2}{\left[\frac{{J}_{1}(us)}{us}\right]}^{2}{s}^{2}dsdu\,=\,{\displaystyle{\int} }_{{u}_{1}}^{\infty }\frac{1}{{u}^{3}}{\displaystyle{\int} }_{0}^{2u}{\left[{J}_{1}(\xi )\right]}^{2}d\xi du=O\left(\frac{{\rm{In}}{{u}}_{1}}{{{u}}_{1}^{2}}\right)\\ {\displaystyle{\int} }_{{u}_{1}}^{\infty }{I}_{Confocal}(u)du\propto {\displaystyle{\int} }_{{u}_{1}}^{\infty }{\displaystyle{\int} }_{0}^{2}{\left[\frac{{J}_{1}(us)}{us}\right]}^{2}sdsdu\,=\,{\displaystyle{\int} }_{{u}_{1}}^{\infty }\frac{1}{{u}^{2}}{\displaystyle{\int} }_{0}^{2u}{\left[\frac{{J}_{1}(\xi )}{\xi }\right]}^{2}\xi d\xi du=O\left(\frac{1}{{{u}}_{1}}\right)\\ {\displaystyle{\int} }_{{u}_{1}}^{\infty }{I}_{Two-photon}(u)du\propto {\displaystyle{\int} }_{{u}_{1}}^{\infty }{\displaystyle{\int} }_{0}^{2}{\left[\frac{{J}_{1}(us/2)}{us/2}\right]}^{2}sdsdu\,=\,{\displaystyle{\int} }_{{u}_{1}}^{\infty }\frac{4}{{u}^{2}}{\displaystyle{\int} }_{0}^{2u}{\left[\frac{{J}_{1}(\xi )}{\xi }\right]}^{2}\xi d\xi du=O\left(\frac{1}{{{u}}_{1}}\right)\\ {\displaystyle{\int} }_{{u}_{1}}^{\infty }{I}_{structured}(u)du\propto {\displaystyle{\int} }_{{u}_{1}}^{\infty }{\left[\frac{{J}_{1}(u{s}_{0})}{u{s}_{0}}\right]}^{2}du=O\left(\frac{1}{\sqrt{{{u}}_{1}}}\right)\\ {\displaystyle{\int} }_{{u}_{1}}^{\infty }{I}_{Lightsheet}(u)du\propto {\displaystyle{\int} }_{{u}_{1}}^{\infty }{\exp (-{u}^{2}/2c^{2})}du=O\left(\frac{1}{\exp ({{u}}_{1}^{2}/2)}\right)=O\left(\frac{2}{1+{u}_{1}^{2}}\right)\\ \displaystyle\int_{u_{1}}^{\infty} I_{Line\_Confocal} (u) du\propto\displaystyle\int_{u_{1}}^{\infty}\displaystyle\int_{0}^{2}\left[\frac{J_{1}(us)}{us}\right]^{2}\,dsdu =\displaystyle\int_{u_{1}}^{\infty}\frac{1}{u}\displaystyle\int_{0}^{2u}\left[\frac{J_{1}(\xi)}{\xi}\right]^{2}\,d{\xi}du=\infty \end{array}\right.$$

where *u*_*1*_ is the defocus distance, larger than the depth of focus. And *ξ* is the production of *u* and *s*.

Figure 3e presents the attenuation factor curves for the methods defined by Eq. (7). The graph demonstrates that LiMo achieves a notably greater signal suppression at 2 μm compared to other methods. Without considering physical size constraints and tissue scattering in practical optical systems, the optical sectioning strength ranks in the order as follows: LiMo being the best, followed by point confocal, two-photon (TP), light sheet, structured illumination, and LC is the least effective. The optical sectioning method of off-axis separated detection takes advantage of the modulation in off-axis detection to improve signal extraction and reduce background effectively. Therefore, superior sectioning strength can be achieved.

#### Various reconstruction methods for separated detection

**Fig. 4:**
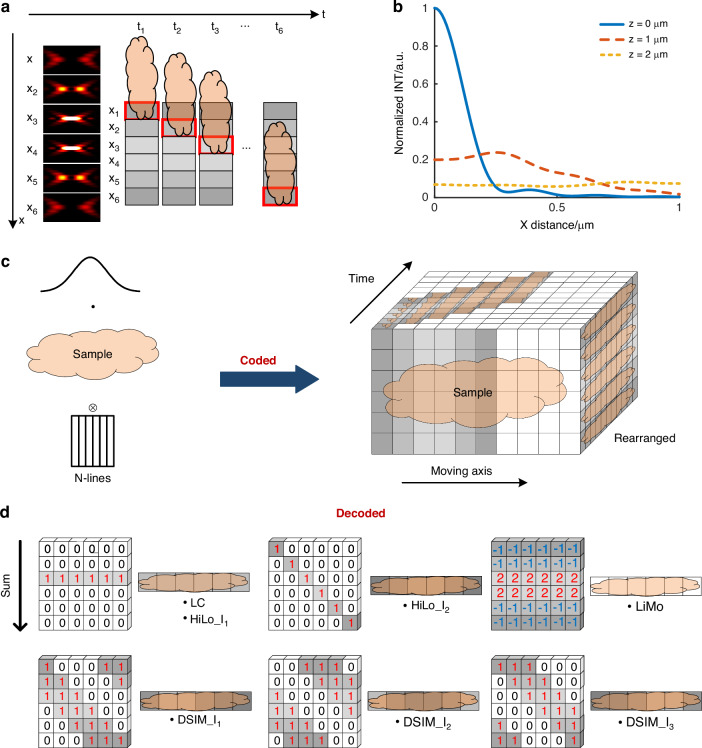
Spatiotemporal multiplexing scanning of separated detection in conjunction with various reconstruction methods. **a** Schematic diagram of spatiotemporal multiplexing scanning. **b** Intensity variation along the off-axis direction and the defocused direction. **c** Schematic diagram of acquiring raw data. **d** Schematic diagram of different reconstruction methods based on raw data

The separated detection method demands multiple scans, which can be time-consuming, especially for weak signals. To enhance the imaging throughput, we can employ spatial-temporal multiplexing. Spatial multiplexing involves simultaneous pixelated detection at multiple off-axis positions to reduce the time caused by additional exposures at a single off-axis position. On the other hand, temporal multiplexing involves conducting various detections of the same position at different times. The multiplexing enables the collection of signals from multiple off-axis positions in a single scan (Fig. 4a).

Moving the sample through the illumination in sequence and employing spatiotemporal multiplexing in separated detection makes it possible to acquire both in-focus and out-of-focus spatial information across the whole sample in a single scan. Moreover, owing to the slit effect of pixelated detection, thick samples are virtually sectioned into thin slices, which can be applied with optical sectioning reconstruction. Such an approach yields superior results by removing problems such as low illumination contrast encountered in the direct processing method when dealing with thick samples. Theoretically, off-axis separated detection has the potential to perform various optical sectioning processes, with different sectioning strengths dependent on the chosen reconstruction method.

Several reconstruction methods are formed based on the capability of off-axis spatiotemporal multiplexing detection to encode both in-focus and out-of-focus information. In detail, the natural modulation in the illumination leads to a difference in intensity distribution along the off-axis direction, which can be described by Eq. (8). When the degree of defocus increases, the system’s capacity to handle high-frequency components diminishes. In contrast, that of the low-frequency components remains unaffected. In other words, natural modulation in illumination rapidly attenuates into uniform illumination when defocusing, as shown in Fig. 4b, which forms the basis for extracting in-focus information^9^.

8 $$PS{F}_{ill}=S(x,y)\otimes {h}_{eff}(x,y,u)=\int {\left|\int \varphi (\rho ){J}_{0}(r\rho )\rho d\rho \right|}^{2}\,dy$$

where *ρ* is the normalized pupil radius, *r* is the radial optical coordinate, and *φ* is the pupil function.

Separated detection also involves splitting the entire PSF of the wide-field detection system into several sub-PSFs based on the degree of off-axis, and these sub-PSFs represent different mixtures of in-focus and out-of-focus information.

9 $$PS{F}_{\det n}={h}_{eff}(x,y,u)\otimes D(x+np,y)={\left|\int \varphi (\rho ){J}_{0}(r\rho )\rho d\rho \right|}^{2}\otimes rect(x+np,y)$$

In Eq. (9), np represents the off-axis distance. As Fig. 4b shows, smaller off-axis distances result in more in-focus information.

To simplify the model, we assume the sample as an impulse function along the optical axis, with its intensity randomly varying with the z-axis position.

10 $$\begin{array}{c}{I}_{n}(x,y)=\mathop{\sum }\limits_{{z}_{0}=0}^{\infty }[PS{F}_{ill}(x,y,{z}_{0})PS{F}_{\det n}(x+{x}_{n},y+{y}_{n},{z}_{0})]\otimes A({z}_{0})\delta (x,y)\\ =\mathop{\sum }\limits_{{z}_{0}=0}^{\infty }A({z}_{0})[PS{F}_{ill}(x,y,{z}_{0})PS{F}_{\det n}(x+{x}_{n},y+{y}_{n},{z}_{0})]\end{array}$$

where *I*_*n*_ and *z*_*0*_ represent the image obtained by the *n*^th^ detector at a position (i.e., *z*_*0*_) on the z-axis, respectively. *A*(*z*_*0*_) is the fluorescence intensity coefficient at *z*_*0*_, determined by the sample.

Moreover, the signal from each sub-detector is the sum of the signals from different depths. The objective lens acts as a low-pass filter with a cutoff frequency that decreases with defocusing. In other words, defocusing decreases response intensity at a specific frequency. As for the natural modulation illumination, when in focus, its response intensity varies with the off-axis distance. With defocusing, it degrades into uniform brightness along the off-axis direction. Therefore, the in-focus component has varying intensities at different off-axis distances, while the out-of-focus component remains consistent, as shown in Fig. 4b.

To utilize encoded information in off-axis separated detection across the whole sample, we can rearrange images detected via spatial-temporal multiplexing based on various off-axis positions (Fig. 4c).

For effective data extraction, a deeper understanding of sub-PSFs is essential. The sub-PSFs from different off-axis positions can be recombined and computed to extract in-focus information. Furthermore, the sum of sub-PSFs is equivalent to the PSF in the traditional coaxial imaging method, expressed in Eq. (11).

11 $$PS{F}_{all}=\mathop{\sum }\limits_{n=-\infty }^{\infty }[{\rm{S}}(x,y)\otimes {h}_{eff}(x,y,u)]\times [{h}_{eff}(x,y,u)\otimes D(x+np,y)]$$

The scanning strategy employed by scanning microscopy allows imaging of a specific position *x* at a given time *t*. Hence, applying modulated illumination at the illumination end is equivalent to introducing a mask at the detection end, which involves arranging and combining the sub-PSFs obtained from off-axis spatiotemporal multiplexed detection. Theoretically, with a sufficient number of off-axis detectors, it becomes feasible to get a set of sub-PSFs as complete as possible, which enables the generation of images in arbitrary patterned modulated illumination by post-processing^15^.

12 $${I}_{image}=\{[{S}_{p}(x)\otimes {h}_{ill}(x)]f(x)\}\otimes {h}_{\det }(x)={h}_{ill}(t)\otimes \{f(t)\otimes [{h}_{\det }(t)\otimes M(t)]\}$$

where *S*_*p*_, *f*(*x*), and *M*(*t*) respectively indicate the illumination pattern, fluorescence distribution of the sample, and the mask in front of the detector.

In summary, the off-axis spatiotemporal multiplexed detection theory allows for flexible modulated illumination using encoded in-focus and out-of-focus information. Additionally, focal plane conjugation methods can also be adopted due to pixelated detection. Therefore, in off-axis separated detection, the PSFs for LC, digital structured illumination microscopy (DSIM), digital hybrid illumination microscopy (DHiLo), and LiMo can be mathematically expressed in Eq. (13). Furthermore, the variations in optical sectioning strengths among these methods^7,16,17^ (in the same system) can be illustrated in Fig. 3f.

13 $$\left\{\begin{array}{l}{Re}cons{t}_{LC}=PSF({v}_{x}+{t}_{3})={I}_{3}\\ {Recons}{{t}}_{DSIM}=abs\left(\mathop{\sum }\limits_{i=1}^{3}[\mathop{\sum }\limits_{i=1}^{6}{M}_{i}(n)PSF({v}_{x}+{t}_{n})]\exp (j\times 2\pi i/3)\right)=\sqrt{5{({I}_{3}-{I}_{1})}^{2}}\\ {Recons}{{t}}_{DHiLo}=HP\{PSF({v}_{x}+{t}_{3})\}+\eta \times LP\{{C}_{S}\times PSF({v}_{x}+{t}_{3})\}={I}_{3}-\gamma LP({I}_{3})\\ {Recons}{{t}}_{LiMo}=2\mathop{\sum }\limits_{i=3}^{4}PSF({v}_{x}+{t}_{n})-\mathop{\sum }\limits_{i=1,2}^{5,6}PSF({v}_{x}+{t}_{n})=\eta ({I}_{3}-{I}_{1})\end{array}\right.$$

where *PSF* denotes the product of illumination and detection PSFs. *η*, *C*_*s*,_ and *γ* are the coefficients in HiLo reconstruction. *HP* and *LP* stand for high-pass filter and low-pass filter, respectively.

As the specific reconstruction methods shown in Fig. 4d, off-axis spatiotemporal multiplexing detection can implement both the focal conjugation and modulated illumination methods employed in coaxial detection. Moreover, with off-axis imaging, the optical sectioning strength is improved as the in-focus and out-of-focus signals are no longer highly overlapped.

**Fig. 5:**
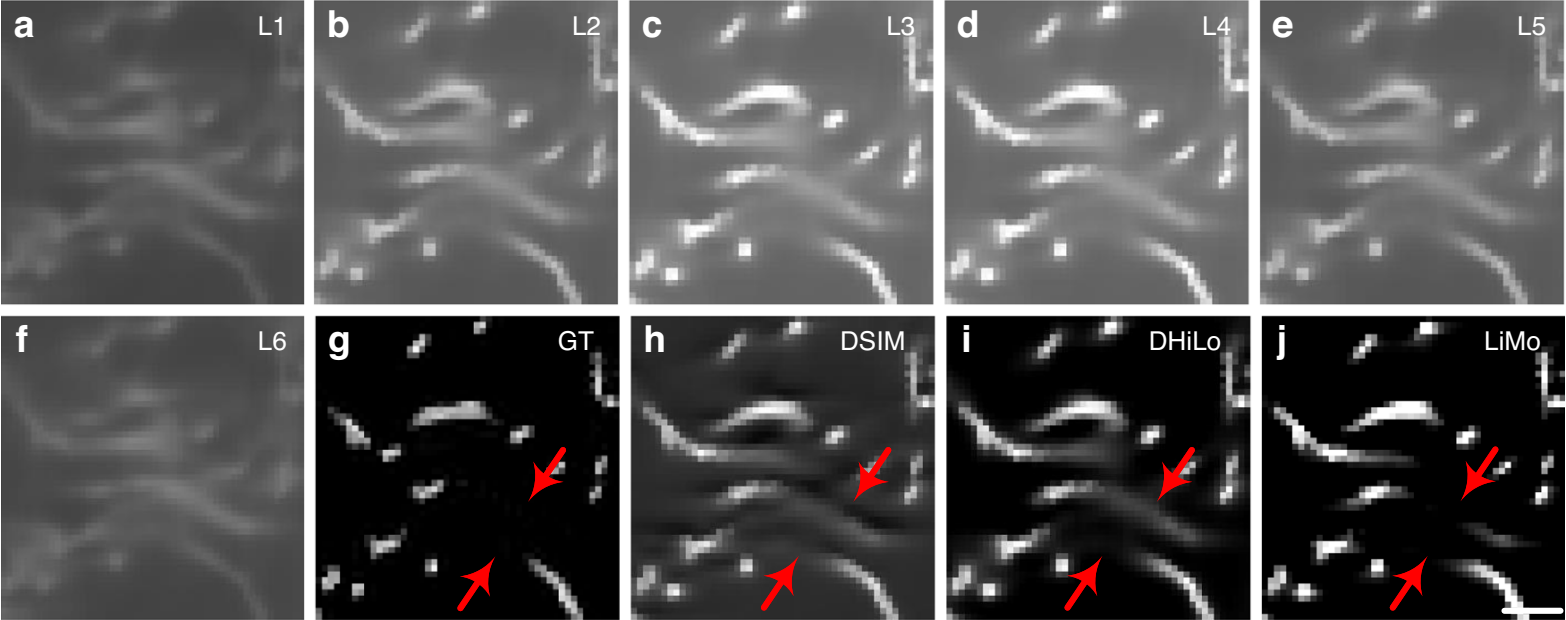
Simulation results of different optical sectioning methods by off-axis spatiotemporal multiplexed detection on the synthetic sample. **a**–**f** Raw data at different off-axis positions. **g** The 512th layer of the synthetic sample. **h**–**j** Reconstructions of g via DSIM, DHiLo, and LiMo, respectively. The red arrows indicate the difference between ground truth and reconstructions. Scale bar: 3 μm in **a**–**j**

To visually illustrate the differences among various optical sectioning methods in the off-axis separation detection system, a random trajectory in a 1024×1024×1024-pixel volume block is generated to simulate the imaging sample. The lateral and axial sampling rate is 0.325 μm and 0.5 μm, respectively. The system has a 20× objective (NA 1.0, XLUMPLFLN 20XW, Olympus) for line-scanning imaging. Combining the linear imaging system model with various decoding methods, we conduct simulations to compare different optical sectioning methods. From the simulation results in Fig. 5, the in-focus signal has high intensity along the central line, while the signal along the edges is nearly indistinguishable from the background. As evidenced by the comparison with the original sample after decoding, LiMo exhibits the highest optical sectioning strength, which retains residual background due to the depth of focus, followed by DHiLo and then DSIM. The images after decoding are superior to those directly obtained via line confocal imaging, indicating that off-axis separated detection can achieve optical sectioning via various methods.

## Development of optical sectioning techniques

As discussed in the previous section, coaxial and off-axis imaging modes employ distinctive optical sectioning methods, leading to diverse techniques. In this section, we present an overview of the development of various optical sectioning techniques with their critical elements summarized in Table 1.

**Table 1:** Summary of optical sectioning techniques

Optical path setting	principle	Technique	Type	R*/ μm	SBR/dB	Through-Put*/ Mpx/s	Speed */ mm^2^/s	Advantages	Disadvantages	Applications
**Coaxial imaging**	Focal plane conjugation	Confocal	Conventional^191^	0.22	12	7.9	0.16	Flexible OS	Low scanning speed	Local fine imaging of biological tissue
			Deep learning^32^	0.12	/	12	0.17	High throughput	Long training time	Organelle interactions
			ISM^192^	0.12	/	1.2	0.08	High resolution	Low scanning speed	Cellular structures imaging
		Spinning disk^193^	/	0.51	/	419	2.8	High throughput	Crosstalk	Intravital imaging
		Line confocal^26^	/	0.42	6.3	4915	27	High scanning speed	Low OS	Whole slide imaging
	Intra-focal excitation	Two-photon	Conventional^58^	1.2	1	7.9	0.16	Deep penetration	High light bleaching	
			Multi-focus^62^	1.4	/	200	0.19	High throughput	Severe tissue scattering	
			Line scanning^69^	0.77	5.5	98	8.5	High throughput	Severe tissue scattering	Intravital imaging
			Wide field^74^	0.61	/	419	9.9 (10X)	Dynamic observation		
		TRIF^80^	/	0.21	/	419	0.11 (100X)	High axial resolution	Depth Limitation	Organelle interactions
	Modulated illumination	SIM	OS-SIM^85^	0.39	12	69	2.1	Low phototoxicity	Illumination pattern sensitive	Large sample imaging
			SR-SIM with PSF engineering^94^	0.09	/	140	0.09 (100X)	High resolution	Manual adjustment	Cellular structures imaging
			TRIF-SIM^80^	0.10	/	70	0.08 (100X)	High OS	Depth limitation	
			3D SIM^96^	0.10	/	28	0.05 (100X)	3D super-resolution with high OS	Strict for optical alignment	
			Scanning SIM^194^	0.10	/	/	/	High quality in modulation	Low scanning speed	
			Deep-learning SIM^105^	0.52	12	419	2.8	Low noise levels	Large training data	Tissue imaging
		HiLo	Conventional^86^	0.41	19	209	2.6	High temporal resolution	Long post-processing time	Intravital imaging
			Line-scanning HiLo^116^	7.5	25	23	/	High scanning speed	Long post-processing time	
**Off-axis imaging**	Mixed detection	Light sheet	SPIM^195^	0.68	3.7	25	1.4	Low phototoxicity	Heterogeneous OS	Clear tissue imaging
			Bessel light sheet^74^	0.68	/	11	0.81	Large thin illumination	Sample size limitation	
			iSPIM^136^	0.52	/	32	6.4	Isotropic resolution	Objective limitation	Clear thick tissue imaging
			siSPIM^153^	0.45	/	248	17	No size limitation	Low energy efficiency	Large, clear, thick tissue
		DSIM^16^	/	0.45	/	53	5.0	High quality in modulation	Redundant acquisition	Whole slide imaging
	Separated detection	DHiLo^17^	/	0.44	/	53	5.0	High robustness	Long post-processing time	Whole slide imaging
		LiMo^7^	/	0.39	26	53	5.0	High OS	Redundant acquisition	Whole brain imaging

*Note that throughput depends on the effective pixel update rate of the camera, which is calculated based on the data reported in the article. Speed is the ratio of imaging area to time, including exposure, data processing, storage, and field-of-view transition time, which is estimated by imaging a 10 mm × 10 mm sample with a field-of-view transition time of 0.15 s. R and Mpx/s represent resolution and Mpixel/s, respectively. SBR represents the signal-to-background ratio. OS represents optical sectioning strength. ‘/’ denotes parameters not reported in the article

### Coaxial imaging

#### Focal plane conjugation

In coaxial imaging systems, confocal microscopy is the most common technique based on focal plane conjugate. First proposed by Minsky, confocal microscopy focuses the illumination light into a tiny point while incorporating a pinhole in front of a photodetector to block out-of-focus signals^18^. The size of the pinhole is adjustable for controlling the sectioning strength and signal-to-noise ratio. Today, confocal microscopy is highly commercialized and has become the gold standard in the field^19,20^. However, current technology has yet to deliver desirable imaging speed^21^. With the fastest scanning device, such as a resonant scanner, a throughput of up to 7.86 Mpixel/s is achievable. Recent developments in focal plane conjugation have been focused on enhancing imaging speed and resolution^22^.

**Fig. 6:**
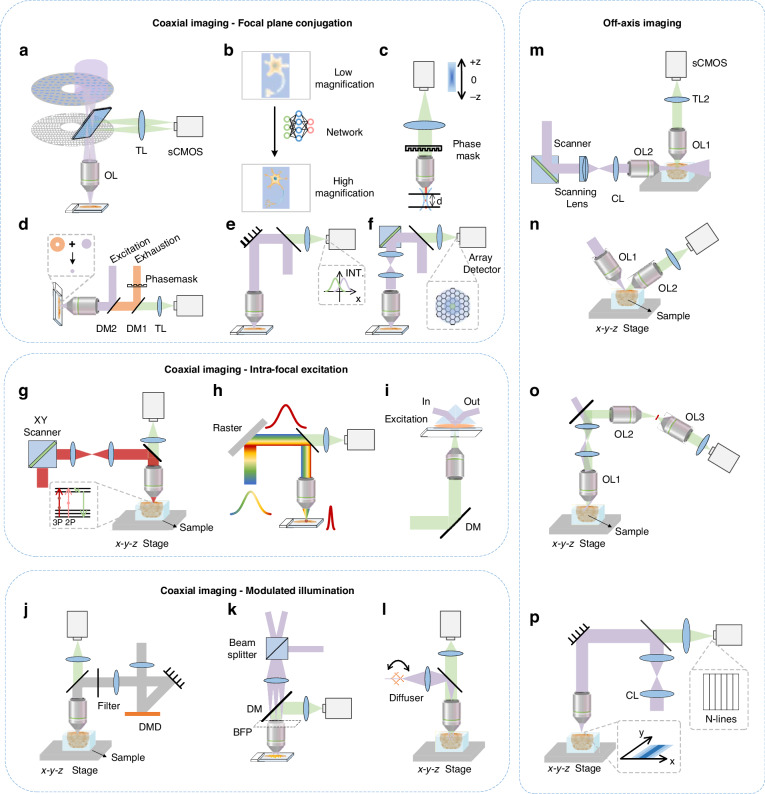
The schemes of recent developments. **a** Spinning disk microscopy. **b** Deep-learning assisted throughput enhancement. **c** PSF engineering-based throughput enhancement. **d** Stimulated emission depletion microscopy. **e** Image scanning microscopy. **f** Fluorescence differential microscopy. **g** Random-access multi-photon microscopy. **h** Temporally focused multi-photon microscopy. **i** Total internal reflection fluorescence microscopy. **j** DMD-based structured illumination microscopy. **k** 3D structured illumination microscopy. **l** Hybrid illumination microscopy. **m** Digitally scanned light sheet microscopy. **n** Open-top light sheet microscopy. **o** Single-objective light sheet microscopy. **p** Line-illumination modulation microscopy. OL, objective lens; TL, tube lens; DM, dichroic mirror; BFP, back focal plane; CL, Cylindrical lens

The technique of multi-focus parallel scanning has been proposed to improve the imaging throughput. The scanning grid can be generated by a spatial light modulator (SLM) at the entrance pupil of the objective or directly by an array of micro-lenses to enable simultaneous excitation and detection of multiple points^23^. Furthermore, with the emergence of line confocal, which adopts line scanning instead of point scanning, the throughput is substantially increased due to the replacement of pinholes with small slits^24,25^. Coupling with specialized line-scan cameras, the throughput can reach 4,915 Mpixel/s^26^. However, the enhancement in imaging throughput comes at the expense of the optical sectioning strength^27^. As such, the technique is applied in slide scanning instead of fluorescence imaging. Spinning disk confocal technology also emerges as a technique that adopts parallel scanning of multiple points to simultaneously generate and capture multiple illumination spots (Fig. 6a). When the rotation speed of the disk matches the exposure time, the imaging speed is equivalent to wide-field imaging, reaching 419 Mpixel/s^28^. However, signal crosstalk between multiple pinholes leads to a decrease in image quality^29^. In recent years, deep learning has been adopted to enhance the imaging throughput of confocal microscopy^30,31^. Specifically, the acquisition process of under-sampled images for further reconstruction into high-resolution images by the network has been accelerated by 16-fold in speed (Fig. 6b)^32^. While deep learning realizes the image fidelity required for biological research, it may fall short of attaining a perfect match to the original image, and the training process can be time-consuming.

Various methods have been adopted to achieve improvement beyond 2D acceleration. For example, PSF engineering enhances imaging speed along the depth axis. By using SLMs or axially distributed reflecting slits, we can image information from different depths without axial scanning (Fig. 6c)^33^. However, PSF engineering requires specialized encoding and lacks optical efficiency. Alternatively, deep learning can extract multi-layer information from single-layer images captured through wide-field microscopy, which further translates to confocal microscopy and improves imaging throughput^34^. Nevertheless, extensive training and sample testing are critical to effectively implementing deep learning.

To improve resolution, stimulated emission depletion (STED) microscopy, which combines confocal microscopy and fluorescence depletion techniques, was developed in 1994 by Hell^35^. In STED, simultaneous illumination with circular excitation and ring-shaped depletion light suppresses a diffraction-limited spot’s edges, improving resolution (Fig. 6d)^36–38^. However, STED causes significant phototoxicity due to the high intensity of the depletion beam^39,40^. A promising solution is to adopt deep learning to convert confocal microscopy images to STED and provide higher image quality and signal-to-noise ratio^41^. Additionally, there is a growing number of efficient solutions to enhance resolution. For example, re-scan confocal microscopy based on back-to-back scanning with an open pinhole can achieve the ideal lateral resolution with a nearly closed pinhole^42^. On the other hand, image scanning microscopy (ISM), which combines confocal microscopy with optical photon reassignment, uses more sub-detectors to correct pixel misalignment between illumination and detection (Fig. 6e)^43–45^. ISM achieves a twofold increase in resolution combined with deconvolution, which can be easily implemented in various microscopes to reduce defocused background and enhance resolution^46–49^. Airy scan technology, an example of multiple-point imaging and ISM reconstruction without additional hardware, has already been applied in commercial microscopes^50^. Similarly, fluorescence differential microscopy (FED), with the use of a point detection array to record signals from the inner ring and the background from the outer ring, has achieved enhanced resolution and signal-to-background ratio by subtraction (Fig. 6f)^51,52^.

#### Intra-focal excitation

In coaxial imaging systems, multi-photon (or two-photon) microscopy is a known technique in intra-focal excitation that employs the nonlinear effect. Multi-photon microscopy selectively excites signals in focus as it requires a higher energy threshold. The power of light, when out-of-focus, is insufficient to generate fluorescent signals, thereby preventing background generation^53,54^. However, multi-photon microscopy is hindered by a similar scanning speed limitation observed in confocal microscopy, which is capped at a maximum rate of 7.86 Mpixel/s.

To increase the imaging throughput, the random-access scanning strategy eliminates the scanning of non-essential areas, thus broadening multi-photon microscopy’s applications in large fields (Fig. 6g)^55–58^. For example, multi-focal excitation has been proposed as a technique^59,60^, which simultaneously detects at different depths or different plane positions within the same plane, thus increasing throughput to speeds in the kHz range to up to 200 Mpixel/s^57,61–65^. We should note that scattering may lead to compromise the image quality. Post-processing calculations based on scattering models can address this issue^66,67^. Also, line-scanning multi-photon microscopy based on time-domain focusing comes to fruition^68^. By temporally focusing a high-power femtosecond laser, the excitation intensity can be axially concentrated by only compensating the grating-induced dispersion at the focal plane, which enables multi-photon excitation for line scanning (Fig. 6h)^69,70^. Though the throughput of line-scanning multi-photon microscopy can reach 97.7 Mpixel/s, it is achieved at the expense of inferior imaging quality caused by tissue scattering^71,72^. Wide-field multi-photon microscopy represents another technique for long-term in vivo observations. Such a technique employs time-domain focusing within a wide field of view; nevertheless, it remains susceptible to tissue scattering and severely compromised axial resolution and thus is often implemented along with other optical sectioning techniques or image post-processing algorithms^73–75^.

In addition to the vulnerability to tissue scattering, multi-photon microscopy is inevitably costly due to the need for high pulse-energy lasers. Total internal reflection fluorescence (TIRF) microscopy is adopted to mitigate the challenge while enhancing optical sectioning strength^76,77^. In TIRF microscopy, incident light undergoing total internal reflection generates an evanescent wave for illumination, which propagates on the surface with a depth of a few tens to hundreds of nanometers (Fig. 6i). With the high axial resolution and optical sectioning strength yielded by the evanescent wave that selectively excites signals from the surface, the technique is well suited for observing interactions between cellular organelles^78,79^. However, the observation via the TRIF microscopy is confined in proximity to the cell membrane due to the limited depth of illumination. Adjusting the angle of incidence can extend TIRF microscopy to observe events up to around one micron beneath the cell membrane, hence having more considerable potential in future applications^80^.

#### Modulated illumination

In coaxial imaging systems, SIM prevails over other techniques in optical sectioning by projecting a periodic structured illumination pattern onto the focal plane and rapidly attenuating with defocusing. After capturing images with three phases, the in-focus information can be extracted, followed by reconstruction to eliminate the structured pattern^81–83^. Neil first proposed the concept in 1997^5^. Then, the technique transitioned from a moving grating to a fast-switching digital micromirror device (DMD) in structured illumination microscopy, significantly improving imaging speed and throughput (Fig. 6j)^84–87^. However, challenges remain for traditional 2D structured illumination microscopy to deliver super-resolution and optical sectioning simultaneously^88–91^. Moreover, the quality of reconstruction is sensitive to variations in the phase of the illumination pattern^12,92,93^.

PSF engineering reduces the normalized optical transfer function at zero frequency in super-resolution microscopy to address the issue^94^. However, manual adjustment of certain parameters is required depending on the imaging sample to obtain optimal results. As such, a different approach combining SIM with TIRF microscopy is proposed to achieve excellent optical sectioning strength^95^. In the x-y plane, 2D super-resolution structured illumination is employed to achieve high lateral resolution; nevertheless, the depth of observation is limited. While transitioning from 2D structured illumination to a 3D version with multiple-beam interference may solve axial deficiencies in super-resolution reconstruction (Fig. 6k)^96–98^, such an approach cannot be realized without a complex optical system and the associated design challenges.

Being more robust against sample or system imperfections, point-scanning SIM employs pixel reassignment-based reconstruction with modulation in laser power during scanning or at the detection end^99,100^. Additionally, point-scanning SIM enables the integration of multi-photon microscopy with SIM^101,102^, and line-scanning SIM mitigates the reduction in imaging throughput associated with point scanning^103,104^. However, multiple acquisitions at the same sample position are still required, resulting in redundant data collection.

Unlike the SIM, which typically requires only a minimum of three repeated captures, 3D structured illumination requires 15 raw images for reconstruction, which is time-consuming^96^. Although deep learning can be introduced to minimize the number of captures, much training data is still required and may not be universally applied to all samples^105,106^. Furthermore, multiplexing methods for achieving optical sectioning in a single capture may complicate the system^107,108^. Additionally, hybrid illumination microscopy (HiLo), first proposed by Mertz in 2006, reduced the number of captures from three to two through frequency-domain filtering and only extracted in-focus information (Fig. 6l)^109–112^. For rapid imaging of living samples, a DMD can be employed to realize a swift switch between two illumination patterns on the detector^86,113^. Moreover, the integration of line-scanning HiLo technique and time-domain focusing facilitates the realization of two-photon HiLo, thereby creating more tremendous application potentiality and imaging utility for larger samples^114–116^.

### Off-axis imaging

#### Mixed detection

Off-axis mixed detection, exemplified by light sheet microscopy, employs two objectives, one for detection and another for vertical illumination, to confine the lighting within a relatively small depth. However, the propagation properties of Gaussian beams make it difficult to achieve uniform resolution and optical sectioning strength across the entire field of view. Digitally scanned light sheet microscopy settled the uniformity issue by employing galvanometers for reciprocal sweeping of the illumination beam (Fig. 6m)^117,118^. The galvanometer can also be integrated with modulated illumination or a rolling shutter mode camera to further improve the optical sectioning strength and SNR^119–122^. Similarly, the sectioning strength and field of view are inversely related due to the constraints on the Gaussian beam thickness and transmission distance^123,124^. As such, methods such as multi-view imaging or scanning the illumination across the field are adopted to extend the field of view substantially^125–129^. However, the effectiveness can be affected by sample rotation or the capabilities of the scanning device. Using non-diffracting beams, such as Bessel or Airy beams, enables a larger field of view while maintaining optical sectioning strength^130–134^.

Open-top light sheet microscopy takes an inverted configuration to eliminate the limitations. Such a method involves inclining the objectives and maintaining their perpendicular arrangement (Fig. 6n)^135–140^. The tilted setup in the open-top light sheet microscopy enables nearly isotropic resolution^141,142^. Also, the design overcomes limitations in working distance and imaging area, allowing for high-resolution imaging of larger samples. As the working distance of the objective mainly affects the depth of imaging, which is also constrained by the optical transparency of samples, it typically does not exceed a few hundred micrometers^143–145^. However, the image quality declines with increasing depths due to scattering and the propagation of the light sheet. Additionally, the tilted setup of the open-top light sheet microscopy reduces the spatial distance between the illumination and detection objectives. Therefore, the NA of both objectives is limited, which imposes constraints on the resolution and optical sectioning strength of the system^146,147^. The single-objective light sheet microscopy addresses the mutual constraint by using the same objective for both illumination and detection (Fig. 6o)^148–150^. Techniques like beam scanning or remote focusing assist in detecting the tilted illumination plane^151–153^. The single-objective light sheet microscopy allows imaging of large, thick tissues without geometric constraints, significantly improving imaging throughput. However, the single-objective tilted illumination reduces optical efficiency. Moreover, multi-field imaging and deep learning are used in light sheet microscopy to further broaden applications of light sheet microscopy^154–156^.

Lastly, the lattice light sheet, achieved by modifying the pupil plane of the objective to create structured illumination, enhances resolution beyond the diffraction limit when combined with super-resolution reconstruction^157–160^. However, implementing the technique inevitably involves the conflict between working distance and optical sectioning, posing challenges for high-resolution imaging of large biological samples.

#### Separated detection

In off-axis imaging systems, separated detection methods utilize the Gaussian beam’s properties to extract in-focus information and enhance optical sectioning strength. LiMo is preferred over confocal microscopy in achieving optical sectioning strength as it performs line scanning without additional modulation devices (Fig. 6p)^7^. While suitable for high-resolution and high-optical sectioning imaging, LiMo requires a minimum of two samplings of the same position, thereby compromising the throughput of the camera.

Similarly, with off-axis separated detection, researchers have successfully implemented SIM and HiLo techniques in a single scan with line scanning^16,17^. Both methods rely on off-axis detection to record the modulation of the illuminating PSF, which can be further processed to enhance optical sectioning strength. Moreover, in off-axis spatiotemporal multiplexing detection, HiLo can be integrated with temporal focusing for large deep-tissue imaging^70^.

As of today, as more optical sectioning methods based on off-axis separated detection evolve, there is also a growing presence of diverse technologies^161,162^. The off-axis separated detection separates the PSF in coaxial detection to record the information of illumination modulation, thus enabling the implementation of various sectioning methods based on the same system. Furthermore, as the spatiotemporal multiplexing technique substantially enhances the imaging throughput and expands the possibility of applications, further development in optical sectioning methods is expected to unfold.

## Comparison of different optical sectioning methods

About application scenarios, we can characterize optical sectioning technologies according to their respective optical sectioning strength, resolution, throughput, robustness, weak signal detection, post-processing speed, penetration depth, and optical safety. In this section, we will provide an overview and comparison across several representative techniques, including those for coaxial systems such as point confocal microscopy, spinning disk microscopy, LC, multi-photon microscopy, SIM, and HiLo, as well as those for the off-axis system such as light-sheet microscopy, DSIM, DHiLo, and LiMo.

### Background suppression

Based on the optical sectioning model mentioned earlier and the assumption of ideal illumination, the optical sectioning strength of specific techniques can be represented by the FWHM of Eq. (14), as shown in Fig. 6a.

14 $$I(u)=\left\{\begin{array}{l}\int {T}^{2}(s,u)jinc(\beta s)sds\,{\rm{Confocal}}\\ \int {T}^{2}({s}_{x},0,u)\sin c(\beta {s}_{x}/\pi )d{s}_{x}\,{{\rm{Line}}\,{\rm{Confocal}}}\\ \int T(s/2,u/2)T(s,u)sds\,{\rm{Two}}-{\rm{photon}}\\ \exp (-{u}^{2}/2{c}^{2})T({s}_{x},{s}_{y},u)\delta ({s}_{x},{s}_{y})\,{{\rm{Light}}\,{\rm{sheet}}}\\ \int \delta ({s}_{x}-{{\rm{s}}}_{0})T({s}_{x},0,u)d{s}_{x}\,{{\rm{Structured}}\,{\rm{illumination}}}\\ \int {T}^{2}({s}_{x},0,u)\sin c(\beta {s}_{x}/\pi )[1-\,\cos (2\beta {s}_{x})]d{s}_{x}\,{{\rm{Natural}}\,{\rm{illumination}}}\end{array}\right.$$

where the light sheet microscope employs a 20× detection objective (NA 1.0, XLUMPLFLN 20XW, Olympus) and a 25× illumination objective (NA 1.0, XLSLPLN25XGMP, Olympus) with a working distance of 8 mm. Theoretical analysis indicates that light sheet microscopy has the highest optical sectioning strength. Based on natural modulation, LiMo achieves stronger sectioning strength than confocal and SIM due to the high modulation frequency. Multi-photon microscopy is slightly inferior to confocal microscopy due to the unconstraint in detection. Spinning disk confocal microscopy suffers from poor performance in background suppression owing to the crosstalk between pinholes. In conclusion, light sheet microscopy exhibits the strongest optical sectioning strength, followed by LiMo and confocal microscopy. SIM and HiLo come next, followed by multi-photon microscopy and then LC. Spinning disk confocal microscopy exhibits weaker sectioning capabilities. However, the limitations in modulation frequencies affect the performance of SIM and HiLo.

Additionally, the optical sectioning strength is weakened due to inaccurate phases. In practice, the performance of light sheet microscopy can be compromised by tissue scattering^163^. It should be noted that the illumination objective is constrained by the balance between the working distance and optical sectioning strength, particularly when imaging large samples at the expense of illumination NA.

### Resolution

When evaluating traditional methods without super-resolution, the constraints on the illumination and detection ends determine the resolution, represented by the FWHM of the PSF. Considering the detection end (slit or pinhole as ideal), the PSF of both the focused mode with constraints on illumination and detection and wide-field mode can be expressed by the following equation, as shown in Fig. 6b.

15 $$\left\{\begin{array}{l}PS{F}_{foused}={{h}_{eff}}^{2}\\ PS{F}_{wide}={h}_{eff}\end{array}\right.$$

The resolution of confocal microscopy with an ideal pinhole is 1.41 times higher than that of wide-field microscopy. However, the size of the pinhole does not diminish infinitely in reality, hence limited improvement in resolution. Taking the average of the resolutions in the *x* and *y* directions to measure the overall resolution, the highest to the lowest resolution is as follows: confocal and spinning disk, LiMo and LC, light sheet microscopy, HiLo, and SIM. In multi-photon absorption, molecules absorb the combined energy of multiple photons simultaneously, allowing excitation with lower-energy photons compared to single-photon absorption. Hence, multi-photon microscopy, with longer wavelengths and unconstrained detection, exhibits lower resolution. Also, the light sheet microscopy may have uneven resolution along the direction of light propagation due to the non-uniformity of the Gaussian beam.

### Speed

In general, the size of the imaging area dictates the imaging speed due to the difference between mosaic scanning and line scanning. Mosaic scanning requires multiple stops for stitching large fields of view, while line scanning has continuous motion along one direction. The acquisition time for both types of scanning is as follows:

16 $$\left\{\begin{array}{lll}{T}_{m} &=& \left({t}_{m}+\sqrt{\frac{4M}{a}}\right)\frac{{L}^{2}}{{M}^{2}}-\sqrt{\frac{4M}{a}}\\ {T}_{s} &=& \frac{{L}^{2}{t}_{s}}{pM}+\left(\frac{L}{M}-1\right)\sqrt{\frac{4M}{a}}\end{array}\right.$$

In Eq. (16), *T*_*m*_ and *T*_*S*_ represent total imaging time via mosaic scanning and strip scanning; correspondingly, *t*_*m*_ and *t*_*s*_ represent exposure times, *a* represents the acceleration of the translation stage, *M* represents the side length of a single field of view, *L* represents the side length of the sample, and *p* represents the sampling size.

17 $$\left\{\begin{array}{l}{T}_{m\_spot}=1.45{L}^{2}-0.12\\ {T}_{m\_disk}=0.28{L}^{2}-0.12\\ {T}_{m\_wide}=0.27{L}^{2}-0.12\\ {T}_{s}=0.09{L}^{2}+0.18L-0.12\end{array}\right.or\,\left\{\begin{array}{l}{T}_{m\_spot}=1.2{L}^{2}\\ {T}_{m\_disk}=2.3\times {10}^{-2}\times {L}^{2}\\ {T}_{m\_wide}=2.3\times {10}^{-2}\times {L}^{2}\\ {T}_{s}=\,\max (3.9\times {10}^{-4}\times L,2.0\times {10}^{-2}\times {L}^{2})\end{array}\right.$$

Using a 20× objective (NA 1.0, XLUMPLFLN 20XW, Olympus) with *M* = 0.67 mm and an acceleration of 200 mm/s², assuming a width of 2,048 pixels in a single field of view, exposure times can be determined as follows. In wide-field imaging, the camera readout speed limits the fastest frame rate, reaching up to 121 fps, meaning *t*_*m*_ = 8.3 ms. In line scanning imaging, the frame rate can reach 300 kHz using a specialized camera (8 bit, 16 K, ML-HC-16K10T, DASLA), which gives *t*_*s*_ = 0.4 μs. In point scanning, due to the frame rate of the detector surpassing that of scanning devices, the scanning speed *v* determines the total exposure time *t*_*m*_ for a single field of view, *t*_*m*_ = 2,048 × 2,048/*v*. With high-speed scanning mirrors employed in Olympus FVMPE-RS, FV3000RS, the speed can reach 30 fps for a 512 × 512-pixel region, corresponding to *t*_*m*_ = 533 ms. In spinning disk confocal microscopy, imaging a 2,048 × 2,048 area can achieve a speed of 100 fps in Dragonfly 202, ANDOR, resulting in *t*_*m*_ = 10 ms. In small imaging areas, all methods employ scanning devices to scan the beam, avoiding the time consumption for moving the translation stage. Therefore, the imaging time for small areas is often limited by camera throughput or scanning speed. To sum up, the imaging time of small and large areas can be mathematically expressed by Eq. (17).

**Fig. 7:**
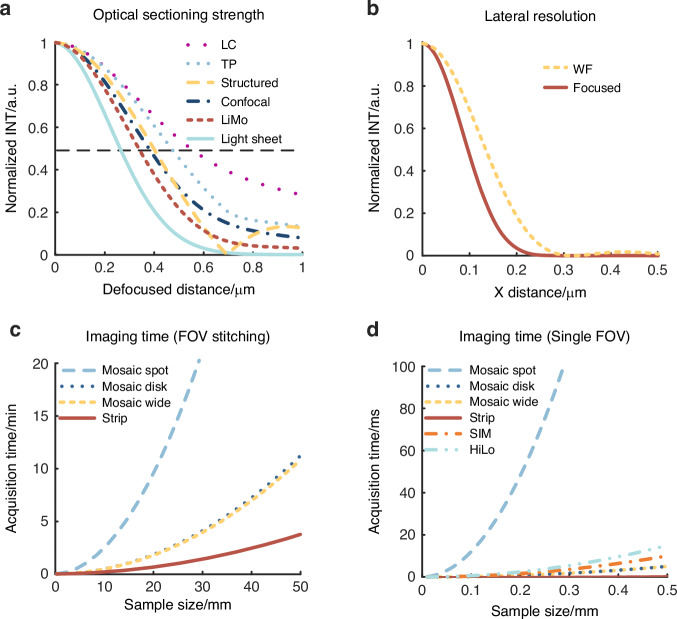
Curves of performance metric. **a** Curves of axial responds via different methods, indicating optical sectioning strength. **b** Curves of lateral PSF, indicating resolution. **c**, **d** Imaging time varies with sample size via different scanning modes when the sample size is larger than the field of view and smaller than the field of view, correspondingly. “TP” and “WF” represent two-photon and wide-field microscopy, respectively

When the sample size *L* changes, the curves shown in Figs. 7c and 7d can be plotted. Line scanning is faster than mosaic scanning in large and small areas due to the high throughput of line-scan cameras. As HiLo and SIM require multiple exposures, longer exposure times and a slightly slower throughput are expected. Similarly, multi-line scanning in LiMo slightly reduces the speed compared to the maximum throughput of line-scan cameras when the maximum throughput is not measured in multi-line working mode. In conclusion, LC, LiMo, and light sheet microscopy enjoy the highest speed, followed by spinning disk confocal microscopy. HiLo and SIM have slower speeds, while point confocal and two-photon microscopy have the lowest speed.

### Robustness

The primary factor to be considered is the impact of the stability of the illumination beam and modulation pattern on the imaging process. Multi-photon and HiLo microscopy, unaffected by the shifting in the beam or variations in the modulation pattern, demonstrate the highest stability. Point confocal, SIM, LC, light sheet, and LiMo microscopy are following, as they are under the influence of at least one factor. For point and line confocal microscopy and LiMo microscopy, precise alignment of beam and aperture positions is required to maintain the SNR and image accuracy. For SIM, accurate phase modulation is essential to avoid striped artifacts in the reconstructed image. Light sheet microscopy requires precise alignment of illumination and detection focal planes to maintain SNR^164^. Whereas, spinning disk confocal microscopy exhibits the lowest stability owing to the requirement for precise perpendicular incidence and alignment with the detector^165^.

### Weak signal detection

SNR is a critical metric for evaluating the ability to detect weak signals via different techniques. Assuming that the images produced by each method are equal and denoted by *“f”*, the SNR in a single image can be calculated as the ratio of the image’s mean to its standard deviation.

18 $$SNR=\left\{\begin{array}{l}\frac{\sqrt{S}f}{\sqrt{{S}_{0}\mathrm{var}(f)}}\,{\rm{Confocal}}\,\&\, {{\rm{Line}}\,{\rm{Confocal}}}\\ \frac{f}{\sqrt{\mathrm{var}(f)}}\,{\rm{Two}}-{\rm{photon}}\,\&\, {{\rm{Light}}\,{\rm{sheet}}}\\ \frac{\sqrt{{C}_{LiMo}}f}{\sqrt{\mathrm{var}(f)}},{C}_{LiMo}=2^\ast \mathop{\sum }\limits_{i=3}^{4}m(i)-(\mathop{\sum }\limits_{i=1}^{2}m(i)+\mathop{\sum }\limits_{i=5}^{6}m(i))\,{\rm{LiMo}}\\ \frac{{C}_{SIM}\sqrt{3}f}{2\sqrt{\mathrm{var}(f)}},{C}_{SIM}=[m(3)+m(4)-m(1)-m(6)]/k\,{\rm{SIM}}\\ \frac{f}{\sqrt{{C}_{HiLo}\mathrm{var}(f)}},{C}_{HiLo}=\frac{\sqrt{\mathrm{var}(m(i))}}{\sqrt{2}}/[\mathop{\sum }\limits_{i=1}^{6}m(i)/6]\,{\rm{HiLo}}\end{array}\right.$$

Therefore, we express the SNR for various optical sectioning techniques as Eq. (18), where *S*_*0*_ represents the area of the illumination spot, *S* represents the area of the pinhole, *k* is the normalization coefficient, *m*(*i*) is the modulation of the *i*^th^ line, and *var()* represents variance. When calculating the SNR for HiLo, it is assumed that the fluctuations of the sample are much smaller than the mean, and the bandpass filter introduces a 3-dB coefficient of $\sqrt{2}$ attenuations, represented as *C*_*HiLo*_ in Eq. (18). By comparing the coefficients in the above equations, HiLo has the highest SNR, followed by LiMo, then two-photon microscopy and light sheet microscopy. SIM has a slightly lower SNR, followed by LC, while point confocal and spinning disk confocal have the lowest SNR^166,167^.

### Post-processing

For reconstruction, confocal, spinning disk, LC, two-photon, and light sheet microscopy have the highest post-process speed for no additional algorithms. LiMo involves a single subtraction step, which accounts for a slightly slower speed. Additionally, SIM reconstruction includes square and square root operations, which is more complex. HiLo reconstruction employs Fourier transforms, resulting in the slowest reconstruction speed.

### Penetration depth

Penetration depth in the imaging process is affected by wavelength and initial optical power, with exponential attenuation as depth increases. In multi-photon microscopy, the penetration depth is directly proportional to the intensity square due to the nonlinear effect. Therefore, the optical power variation with depth in nonlinear and linear microscopy can be expressed as follows^168^.

19 $$\left\{\begin{array}{l}{P}_{linear}={P}_{0}{e}^{-z/ls}\\ {P}_{nonlinear}\propto {({e}^{-z/ls})}^{2}={e}^{-2z/ls}\end{array}\right.$$

where *P*_*linear*_ and *P*_*nonlinear*_ are the illumination power in linear and nonlinear systems at image depth *z*, respectively. *P*_*0*_ is the power at the surface. *l*_*s*_ is a constant distance related to scattering, which is inversely proportional to the illumination angle^169^.

Multi-photon microscopy achieves greater penetration depth with the same initial optical power. Regarding illumination angle, in off-axis separated detection, normal incidence with oblique detection is often employed to split the overall PSF, reducing the influence of illumination angle. In light sheet microscopy, while the illumination angle potentially affects the penetration depth, optical clearing methods or naturally transparent samples effectively neutralize this effect. Therefore, the illumination angle has a minimal impact on penetration depth in off-axis imaging.

### Optical safety

The optical safety is determined by the optical power density and exposure time, which can be calculated using the following equation.

20 $$G=Pt/{S}_{area}$$

where *G* indicates the phototoxicity, *P* is the illumination power, *t* is the exposure time, and *S*_*area*_ is the illuminated area at a certain depth.

The following analysis assumes consistent total energy at the focal plane. As to the phototoxicity of defocused beams on the sample, the spot size increases proportionally with defocusing. Therefore, light sheet microscopy exhibits a decreased phototoxicity as the defocus distance increases due to only one snap in the field of view. Further, the perpendicular optical path confines defocus within a smaller range. Moreover, in scanning microscopy, both the area of illumination and exposure time increase proportionally when defocused, which leads to a consistent level of phototoxicity across defocused and in-focus regions. In the particular case of multi-photon microscopy, excitation only occurs within the focal plane, resulting in no phototoxicity for non-target areas^170–172^. However, since the laser focus still illuminates the focal plane, multi-photon microscopy exhibits relatively strong phototoxicity at the focal region. Considering defocused beams and the area of illumination, light sheet microscopy demonstrates superior optical safety, followed by HiLo and SIM, which require multiple captures.

In comparison, LiMo and line scanning microscopy exhibit lower safety levels. Meanwhile, confocal microscopy exhibits the lowest optical safety, as it causes equivalent bleaching in and out of focus. Due to its unique principles, multi-photon microscopy has higher optical safety than confocal microscopy.

**Fig. 8:**
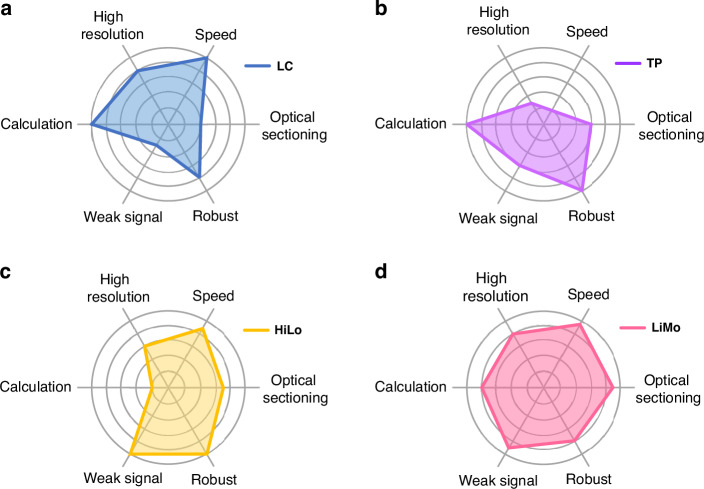
Performance comparison for different metrics. **a**–**d** Comprehensive maps of line confocal, two-photon, HiLo, and LiMo microscopy, respectively. To highlight differences, the dimensionality charts are drawn based on theoretical conditions, from 1 (worst) to 5 (best)

The final score of each technique is calculated based on the performance indicators assigned with a numerical score from 1 to 5, as shown in Table 2. Comprehensive maps of different methods (Fig. 8) indicate that each method has its respective strengths and weaknesses that define its suitability in various application scenarios.

**Table 2:** Rating for various optical sectioning techniques*

	Coaxial imaging						Off-axis imaging			
	Focal plane conjugation			Intra-focal	Modulated illumination		Mixed	Separated		
	Confocal	SD	LC	Two-photon	SIM	HiLo	Light sheet	DSIM	DHiLo	LiMo
Sectioning	3.5	1	2	3	3.5-	3.5-	5-	4	4	4.5
Resolution	5	5	4	1.5	3	3	3	4	4	4
Speed	1	4.5	5	1	4.5-	4.5-	5	5-	5-	5-
Robustness	4	3	4	5	4	5	4	4.5	5	4
Weak signal	1	1	1.5	3	2	5	3	2	5	4.5
Post-process	5	5	5	5	2.5	1	5	2.5	1	4
Penetration	2	2	2	5	2	2	2-	2	2	2
Safety	1	1	3	2+	4.5	4.5	5	3	3	3

*In the table, “-” indicates that the actual effect will be lower than the given rating. “+” indicates that the actual effect will be higher than the given rating. “SD” represents spinning disk microscopy

## Selection of different optical sectioning methods

In biological imaging, the selection of techniques is subject to sample properties, biological state, labeling, and experimental protocols. Firstly, the structure of the sample determines the required resolution; the size and condition of the sample demand different imaging throughputs. While high throughput is rarely necessary for small-scale imaging, low throughput in large-scale imaging will substantially increase imaging time and data acquisition costs. Also, off-axis imaging methods are preferred over coaxial imaging when the sample thickness exceeds tens of micrometers. Secondly, despite the geometric information embedded with biological samples, deeper penetration is required to observe living samples; conversely, optical clearing provides sufficient depths for non-living samples. Thirdly, the signal intensity determined by labeling also dictates the selection of the most optimal imaging methods. Achieving an SNR higher than 1 is crucial for distinguishing weak signals, which should be prioritized. Note that the sample’s ability to tolerate high-power laser, or the phototoxicity tolerance, is subject to its biological state and the labeling method. Lastly, method selection cannot neglect experimental protocols such as observation duration. For example, longer observation duration calls for higher system stability.

In general, owing to the low SNR caused by small pinholes and diminished contrast in modulation, coaxial optical sectioning methods offer weaker optical sectioning strength, which makes them less preferable than off-axis methods for thick samples. In the subsequent discussion, we will review the strengths and weaknesses of various techniques based on their features, rating results, and analysis, followed by an outline of their respective optimal application scenarios.

Confocal microscopy excels in resolution and optical sectioning, being widely available commercially. However, the low throughput constrains its application in small-scale imaging of fixed samples, such as delicate structures in slices. While the issue can be mitigated substantially via rapid scanning resonant mirrors, imaging live cells remains challenging. This is caused by rapid scanning’s operating requirement for shorter dwell time at each point, hence the higher power densities that exacerbate photobleaching issues. Furthermore, confocal microscopy is less suitable for imaging tissues with a thickness of hundreds of micrometers, despite a common strategy to decrease SNR by reducing the pinhole size to enhance optical sectioning strength. Another issue to be noted is that tissue scattering may block in-focus signals and consequently contribute to the collection of out-of-focus signals.

Being an upgrade from conventional confocal microscopy, spinning disk microscopy allows for higher throughput without reducing the exposure time for each point. Such improvement makes it well-suited for imaging live cells or large-scale, low-resolution imaging, such as stromal cell dynamics^173,174^. However, the out-of-focus information, whose illuminated area increases with the square of the defocus distance, is gathered by adjacent pinholes; in other words, the crosstalk between the pinholes that causes reduced optical sectioning strength is the reason why spinning disk microscopy is less suitable for high-resolution imaging. Moreover, due to the fixed size of the pinholes, adjustment for an optimal balance between SNR and optical sectioning strength is unattainable, which is a limitation when dealing with samples of varying properties.

Line-scanning microscopy combines point scanning in one dimension with wide-field imaging in another. When coupled with a high-throughput line-scan camera, line-scanning microscopy excels in applications such as whole-slide scanning^175–177^. However, compared to point-scanning confocal microscopy, line-scanning microscopy lacks the required resolution and optical sectioning strength to deliver high-quality images for high-resolution or thick samples due to the one-dimensional scanning approach.

Multi-photon microscopy distinguishes itself by its superior penetration capabilities in deep tissue observation. Multi-photon microscopy is an indispensable technique for imaging animals through the skull, cranial, or other windows in vivo, as it allows the observation of biological activities of single cells and cell interactions through functional labeling, such as calcium imaging^178–181^. Thanks to the absence of additional small apertures in front of the detector, multi-photon microscopy offers robust stability for long-term observation, such as monitoring changes in transplanted tumor tissues over time. Moreover, the long-term survivability of the sample is superior due to lower phototoxicity compared to point confocal microscopy, as the excitation is only present in focus. However, based on point scanning, multi-photon microscopy leads to slow imaging speed and limited high-frequency capture. While parallel scanning methods^63^ have been proposed, issues such as reducing signal crosstalk and shortening post-processing time remain challenging.

With high throughput and optical sectioning strength, SIM is suitable for detailed imaging of thin samples over a large area such as muscle tissue^182^. However, in thick samples, the tissue background reduces the contrast of the structured illumination and the accuracy in phase alignment of the three images. This weakens optical sectioning strength and striped artifacts in the reconstructed images. Additionally, weak signals are a concern as the nonlinear reconstruction can amplify noises, and in vivo imaging, the reconstruction algorithm is susceptible to motion artifacts. Moreover, capturing three images per field of view lowers the frame rate, which is not a feature suited to capture rapid motion. As such, HiLo microscopy represents a practical approach to address the issues as it only requires one uniformly illuminated image and one non-uniformly illuminated image for dynamic captures, such as fast calcium imaging^183,184^. HiLo also exhibits higher robustness due to its tolerance in phase accuracy and higher SNRs for the extraction of weak signals thanks to its reconstruction algorithm that incorporates bandpass filtering. It should be noted that the algorithm involving Fourier transforms and inverse Fourier transforms increases computational complexity and reconstruction time. As such, HiLo is less effective in tracking the significant motion in live samples.

Light sheet microscopy possesses high optical sectioning strengths thanks to the perpendicularity between the illumination and detection axes, which makes it suitable for thick samples. However, it has limitations in several aspects. For example, the short working distance of a high-NA objective is not ideal for large-volume sample imaging. Also, the attempt to achieve isotropy and higher imaging throughput by tilting the objectives can impose constraints on the geometric dimensions of the two objectives and consequently compromise the NA of the illumination objective. Therefore, light sheet microscopy is more suitable for large-scale, non-fine imaging, especially when combined with optical clearing technology to maximize the imaging throughput^185,186^. Although optical clearing allows the light sheet to penetrate deep tissues, even in transparent samples, tissue scattering still alters the characteristics of the light sheet by turning it into a thicker sheet. In practice, the optical sectioning strength of light sheet microscopy is impeded by tissue scattering and lower NA of the illumination objective as a result of weak signals submerged in the background; nevertheless, it remains an effective method due to high throughput and low phototoxicity which are particularly advantageous in biological applications for live imaging of transparent organisms, such as observing zebrafish heartbeats or imaging complete embryos.

**Fig. 9:**
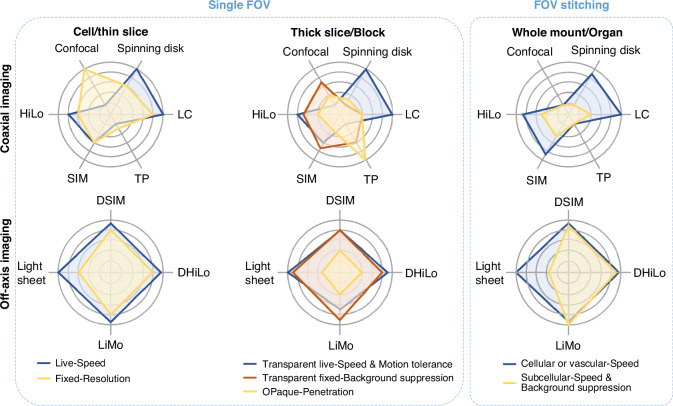
Recommended solutions for different applications. Based on the analyses above, we establish a selection process for different applications based on the sample geometry and specific needs in Fig. 9. In the category of cell samples or even thin slices, most optical sectioning methods are suitable due to the low background signals. The experimental plan depends on specific needs, like speed for live imaging, resolution for fixed samples, and system accessibility. In the category of localized observation in thick samples or blocks, for transparent live animals like zebrafish, where speed and motion resistance are more critical than sectioning strength and thus part of the coaxial and off-axis methods are suitable; for transparent fixed samples like in situ observation of 3D gene expression, due to the density of gene expression and high demands for optical sectioning and resolution, off-axis imaging systems have more advantages; for opaque samples, two-photon microscopy offers a distinct advantage in penetration depths. In whole-mount or organ imaging, field of view (FOV) stitching is employed to cover large areas, making imaging speed equally crucial to other requirements. For imaging single neuronal morphology with axonal resolution through the whole brain, with millimeter dimensions and sub-micrometer resolution, off-axis separated detection with line scanning is preferred for its high optical sectioning and throughput, surpassing light sheet microscopy with NA limitations in resolution. For mapping cell distribution or vessel networks with large volumes and microscale structures, throughput is prioritized over optical sectioning and resolution, making both on-axis and off-axis strip scanning imaging methods suitable. In summary, the coaxial system is comparable to the off-axis system in imaging thin samples with low background signals or thick samples with details to be resolved far bigger than the resolution limit, where the coaxial system is preferred due to its popularity and accessibility. However, the off-axis system is indispensable in imaging thick samples with detailed features close to the resolution limit

Off-axis separated detection methods are particularly advantageous in imaging large and thick samples, as opposed to all other methods mentioned above. Its uniqueness lies in its ability to achieve throughput sectioning concurrently by combining line scanning with different imaging principles at different off-axis positions. Various techniques can be conducted based on off-axis separated detection. LiMo, with the best optical sectioning strength via linear reconstruction, is especially suitable for imaging thick samples such as whole-brain imaging. However, LiMo is sensitive to the position of the illumination spot, which may lead to a reduced SNR and even potential signal loss. For this reason, long-term imaging over several weeks without optical path adjustments remains challenging. Moreover, LiMo is suboptimal for in vivo imaging, as the stability of the illumination spot is under substantial impact from biological respiration and heartbeat. In comparison, DHiLo provides better stability while offering less optical sectioning strength. DHiLo also achieves a higher SNR due to the filtering operations in reconstruction and robustness, which is ideal for live imaging. However, DHiLo has a slower reconstruction speed, hence longer waiting times for real-time viewing. On the other hand, DSIM is a relatively balanced imaging technique that exhibits favorable tolerance to small shifts of the line spot over long-term observations with only a slight addition to the reconstruction time. Nevertheless, DSIM’s accuracy in live imaging and optical sectioning strength is below par when compared with DHiLo and LiMo. Therefore, DSIM is suitable for, for example, long observations in brain slices ex vivo^187^ (Fig. 9).

Overall, the diversity in biological samples and experimental goals prompts the need for various imaging techniques. Nevertheless, the switching between systems complicates the operating process. Therefore, the development of one single system capable of implementing multiple optical sectioning methods to select the most optimal approach can significantly simplify the operations. In this regard, off-axis separated detection is a feasible solution as it allows for the implementation of multiple sectioning techniques in laser scanning microscopy.

## The potential for versatile optical sectioning methods using the same system

The off-axis separated detection theory provides a versatile framework for implementing multiple optical sectioning methods in the same optical system. One strategy to achieve high imaging speed is to include a line scanning module in the system. Specifically, the line-scanning off-axis separated detection optical sectioning method is suitable for imaging large samples at high speed. On the other hand, digital modulation at the detection end offers flexible optical sectioning with many available algorithms and resists scattering, achieving higher contrast than coaxial imaging.

To demonstrate the merit over coaxial imaging, we imaged a 50 μm-thick brain slice of a Thy1-GFP M line transgenic mouse (#007788, Jackson Laboratory) with a 1-μm axial step based on a custom-built off-axis line illumination microscope^7^ and wide-field SIM microscope^85^. Both systems have a 20× objective (NA 1.0, XLUMPLFLN 20XW, Olympus) and an sCMOS camera (ORCA-Flash 4.0, Hamamatsu, Japan). For coaxial imaging, in wide-field SIM, the exposure time was set to 30 ms for each capture, with three images taken per field, followed by mosaic scanning to cover the sample area. In wide-field HiLo, a structured illumination pattern was replaced by full illumination for artifact-free wide-field imaging (other settings are the same as the wide-field SIM). For the line-scanning off-axis separated detection, the line-scan camera worked in 8-line subarray mode for different off-axis positions, with an exposure time of 38.9 μs. The stage speed matched the ratio of frame rate to exposure time.

**Fig. 10:**
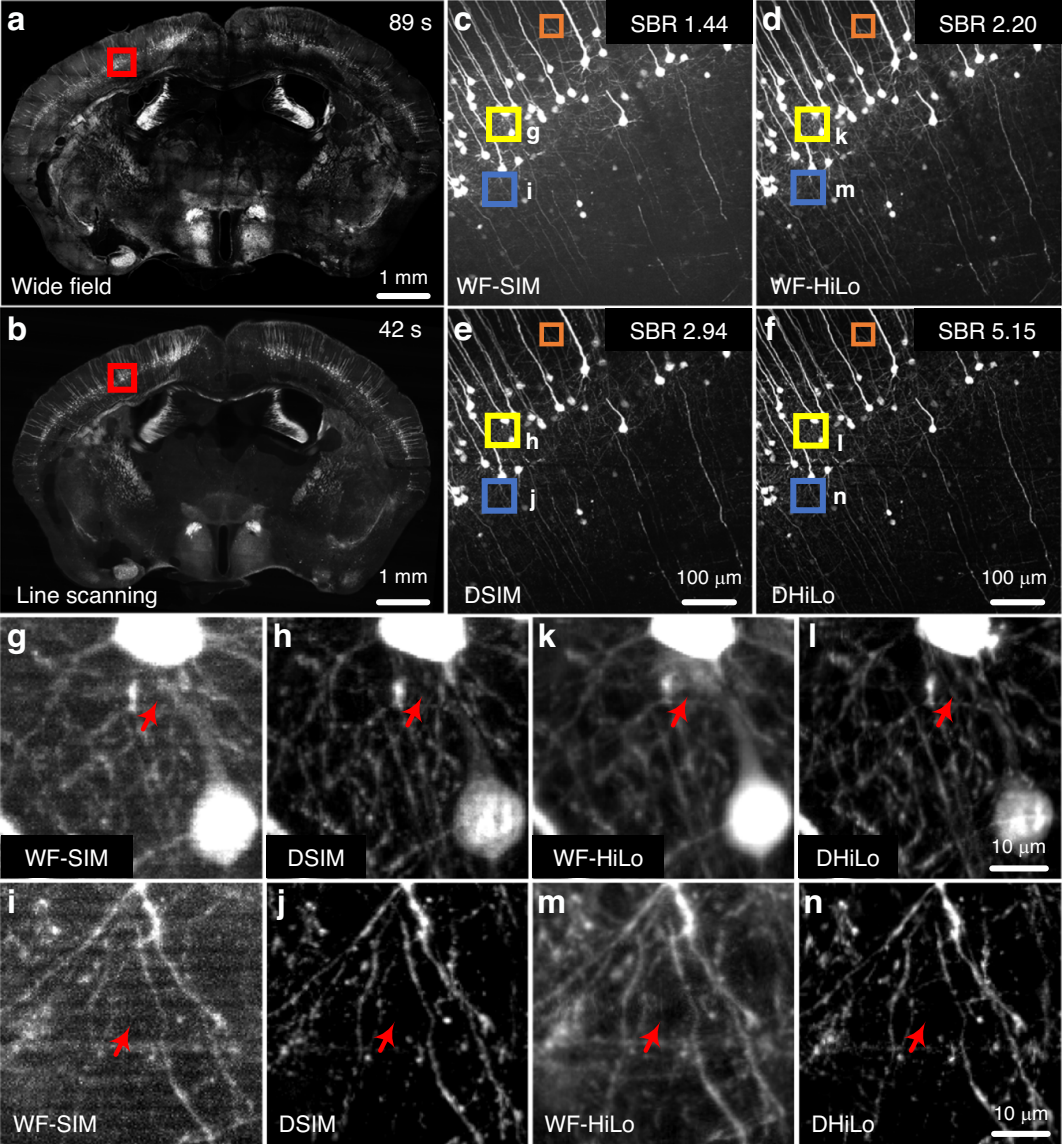
Images of a 50μm thick Thy-1 -GFP M line transgenic mouse brain slice in both line scanning off-axis separated detection and wide-field coaxial systems to compare SIM and HiLo in different detection approaches. **a**, **b** Maximum intensity projections (MIPs) of the mouse brain slice by SIM reconstruction via wide-field (WF) and line scanning imaging approaches, respectively. **c**, **d** WF-SIM- and WF-HiLo-reconstructed enlarged views from the red square in **a**, respectively. **e**, **f** DSIM- and DHiLo-reconstructed enlarged views of the red square in (**b**), respectively. **g**, **h** Enlarged images from the yellow squares in (**c**) and (**e**), respectively. **i**, **j** Enlarged images from the blue square in (**c**) and (**e**), respectively. **k**, **l** Enlarged images from the yellow square in (**d**) and (**f**), respectively. **m**, **n** Enlarged images from the blue square in (**d**) and (**f**), respectively. Scale bar: 1 mm in **a** and **b**, 100 μm in **c**–**f**, and 10 μm in **g**–**n**

Figure 10a and b show the two microscopes’ typical maximum intensity projections (MIPs). The image area is 9.4 mm × 6.3 mm. The imaging time for a single plane was 89 s in wide-field imaging and 42 seconds in line scanning imaging, indicating that the imaging speed of off-axis separated detection was approximately twice faster than traditional wide-field coaxial imaging.

We performed the SIM and HiLo reconstructions of a randomly selected region of interest (ROI) indicated by red squares in Fig. 10a and b, as shown in Fig. 10c–d and Fig. 10e–f. To quantitatively analyze the optical sectioning strength, we calculated the intensity ratio of fiber to background inside the orange squares as signal-to-background ratio (SBR) in Fig. 10c–f. The SBR for wide-field SIM, DSIM, wide-field HiLo, and DHiLo are 1.44, 2.94, 2.20 and 5.15, respectively. The off-axis separation detection system outperforms the wide-field coaxial system in optical sectioning strength.

Further, Fig. 10g and h shows the enlarged views of yellow squares in Fig. 10c and e to reveal that the optical sectioning strength of SIM implemented in the off-axis separated detection system is better than that in the coaxial system. Figure 10i and j shows the enlarged view of blue squares in Fig. 10c and e. The results demonstrate that the SIM in the off-axis separated detection system shows no striped artifacts, while the reconstructed image in the coaxial system suffers from severe striped artifacts. Under HiLo reconstruction, the yellow squares in Fig. 10d and f were enlarged to obtain Fig. 10k–l. The blue squares in Fig. 10d and f were enlarged to obtain Fig. 10m–n. All these results indicate that under the same reconstruction method, the optical sectioning strength in the off-axis separated detection system is superior to that in the coaxial system in regions with more fibers or near somas.

**Fig. 11:**
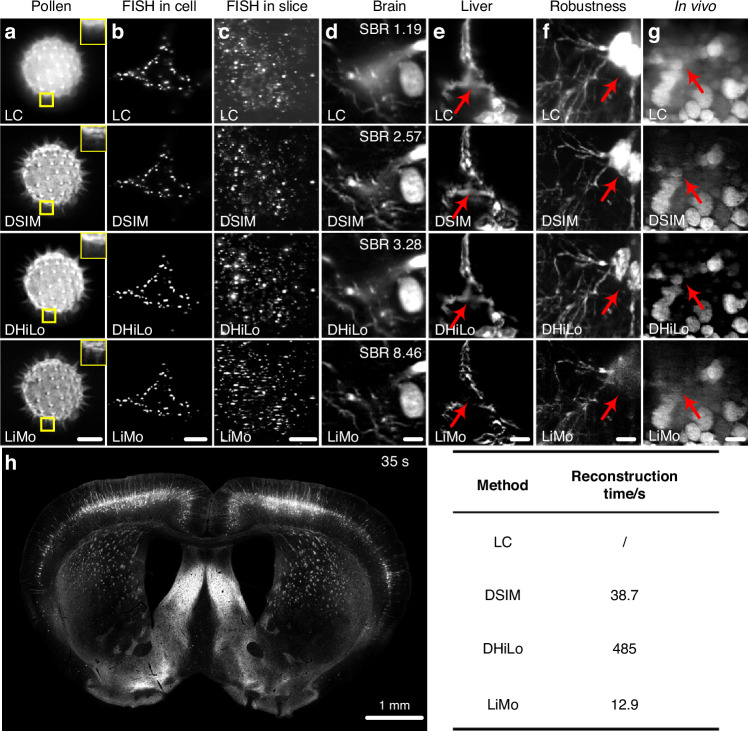
Flexible reconstruction implementations of off-axis separated detection in different applications. Reconstructions of **a** pollen, **b** gene distribution in 3T3 cell, **c** gene distribution in mouse brain slice, **d** mouse brain slice, and **e** mouse liver via different methods. Reconstructions of the **f** mouse brain slice when the linear illumination is titled and **g** mouse liver in vivo via different methods. **h** Coronal plane of the mouse brain and the reconstruction time via different methods. Scale bar: 10 μm in (**a**–**c**), (**e**), and (**g**), 5 μm in (**d**), 20 μm in (**f**), and 1 mm in (**h**)

To demonstrate the flexibility of a single system for versatile methods, we imaged various applications by off-axis separated detection (Fig. 11) with settings matching the line scanning off-axis detection system in Fig. 10. These experiments used 1 μm axial steps and single plane for display. (Note that the pollen and brain slices with tilted line illumination used maximum intensity projections).

Figure 11a shows imaging results of pollen using LC, DSIM, DHiLo, and LiMo. The enlarged views of corresponding yellow squares in each top right corner demonstrate that LiMo yields the best results, particularly in detecting weak pollen spines. Fluorescence in situ hybridization (FISH) is a common technique for visualization of gene expression by dot markings on labeled samples; sample thickness and gene expression density can dictate the selection of optical sectioning methods. Figure 11b displays *Nnat* gene expression in 3T3 cells, where the thin sample and low expression levels make most sectioning methods suitable. However, in imaging *Wscd1* in a 40-μm thick C57BL/6 J brain slice, the background is higher than in cellular FISH imaging (Fig. 11c), necessitating off-axis separated detection sectioning methods. Figure 11d shows the application of different reconstruction methods in the 100-μm thick brain slice, with LiMo demonstrating the highest optical sectioning strength. The SBRs for different methods were measured and calculated to be 1.19, 2.57, 3.28, and 8.46, respectively, confirming the superior background suppression in LiMo. Figure 11e displays the reconstruction results in liver vessels, where all methods provide clear structural visualization. By using line confocal microscopy reconstruction, it is possible to increase the axial sampling interval while still recovering a complete structure and reducing acquisition time. Figure 11f demonstrates the effects of different reconstruction methods when the system is unstable, i.e., when the linear illumination beam has a certain tilt angle. Both DHiLo and DSIM perform well under these conditions. Figure 11g presents the in vivo imaging of mouse liver cells, where a substantial amount of motion interference is due to respiration. The results indicate that only DHiLo achieves satisfactory optical sectioning results.

Figure 11h shows the time required for different algorithms to reconstruct the left mouse brain coronal section. We conducted the test offline using MATLAB 2020a and an Intel(R) Core(TM) i7-9700 CPU @ 3.00 GHz. The table in the bottom left corner shows that the LC is the fastest, while DHiLo is the slowest. All reconstruction methods share the same raw data acquisition time of 35 seconds per layer. Both acquisition and reconstruction time are proportional to the imaging area. Hence, LiMo offers real-time reconstruction, while non-linear algorithms, especially frequency-domain DHiLo, cause delays or require post-processing.

In summary, off-axis separated detection provides excellent performance and a highly flexible possibility for large-sample optical sectioning imaging.

## Summary

The development of optical sectioning techniques has been driven by the pursuit of high-quality 3D structural and functional information for an extensive range of applications in biology. Existing optical sectioning methods, each distinctly developed based on their respective principles and needs, contribute to diverse technologies. Nevertheless, the inherent characteristics of each technique make them suitable under certain circumstances and suboptimal in others. As a result, selecting the most optimal optical sectioning technique, i.e., aligning the sample’s characteristics with the experimental design, becomes paramount.

This review categorizes optical sectioning methods into coaxial and off-axis imaging based on the spatial relationship between illumination and detection axes. As optical sectioning techniques are diverse in features and versatile in applications, we discuss various technologies, offering guidance on their benefits and applications to facilitate the selection of the most optimal technique under multiple scenarios. Due to optical axis configuration, coaxial imaging excels with thin samples but struggles with thick ones.

Though still in the early stage of development, off-axis separated detection has already demonstrated exceptional performance in thick sample imaging. Regardless of the sample thickness, it can maintain a constant mixture ratio of in-focus and out-of-focus at different off-axis positions. Such capability opens the door to implementing flexible optical sectioning techniques and accommodates different needs within the same system. Such a framework also saves the need for expensive system replacements while offering greater flexibility in reconstruction methods. Overall, off-axis separated detection performs unparalleled in large-scale, thick sample imaging. It is ideal for whole-organ studies in biology, especially for acquiring single-neuron morphology with axon resolution across the brain^188–190^.

In the future, we can further expand the application of off-axis separated detection through integration with existing technologies and advancement in reconstruction methods. The system can achieve flexible optical sectioning imaging tens of times faster than confocal microscopy and supports the development of extended functions such as super-resolution, multi-color, and multi-view imaging, which may become a standard in most imaging facilities. Moreover, the off-axis separated detection can also advance materials science by revealing structures and properties. In summary, with their unique optical setup and advantages, the off-axis separated detection systems are expected to be a new direction for future development and become a powerful tool for optical sectioning imaging.

## Data Availability

The data that support the findings of this study are available from the corresponding author upon reasonable request.
